# Allocation and validation of the second revision of the International Staging System in the ICARIA-MM and IKEMA studies

**DOI:** 10.1038/s41408-024-01149-w

**Published:** 2024-11-28

**Authors:** Paul G. Richardson, Aurore Perrot, Joseph Mikhael, Thomas Martin, Meral Beksac, Ivan Spicka, Marcelo Capra, Mattia D’Agostino, Pieter Sonneveld, Kamlesh Bisht, Taro Fukao, Rick Zhang, Keisuke Tada, Christina Tekle, Sandrine Macé, Zandra Klippel, Helgi van de Velde, Philippe Moreau

**Affiliations:** 1 Jerome Lipper Multiple Myeloma Center, Department of Medical Oncology, Dana Farber Cancer Institute, Harvard Medical School, Boston, MA USA; 2 CHU Toulouse, IUCT-O, Université de Toulouse, UPS, Service d’Hématologie, Toulouse, France; 3 Applied Cancer Research and Drug Discovery, Translational Genomics Research Institute, City of Hope Cancer Center, Phoenix, AZ USA; 4 Department of Medicine, University of California, San Francisco, CA USA; 5 Department of Hematology, Ankara University, Ankara, Turkey; 6 General Faculty Hospital and First Faculty of Medicine, Charles University, Prague, Czech Republic; 7 Centro Integrado de Hematologia e Oncologia, Hospital Mãe de Deus, Porto Alegre, Brazil; 8 Struttura Complessa (SC) Ematologia, Azienda Ospedaliero-Universitaria (AOU) Città della Salute e della Scienza di Torino, Turin, Italy; 9 Erasmus University Medical Center Cancer Institute, Rotterdam, The Netherlands; 10 Sanofi, Cambridge, MA USA; 11 Sanofi, Tokyo, Japan; 12 Sanofi, Chilly-Mazarin, France; 13 Hematology Department, CHU Nantes, Nantes, France

**Keywords:** Therapeutics, Cancer immunotherapy

## Abstract

The International Staging System for multiple myeloma recently underwent a second revision (R2-ISS) to include gain/amplification of 1q21 and account for the additive prognostic significance of multiple high-risk features. The phase 3 ICARIA-MM (isatuximab–pomalidomide–dexamethasone vs. pomalidomide–dexamethasone) and IKEMA (isatuximab–carfilzomib–dexamethasone vs. carfilzomib–dexamethasone) studies provide large datasets for retrospectively validating the prognostic value of the R2-ISS in relapsed/refractory multiple myeloma. Of 609 pooled patients, 68 (11.2%) were reclassified as R2-ISS stage I, 136 (22.3%) as R2-ISS stage II, 204 (33.5%) as R2-ISS stage III, 55 (9.0%) as stage IV, and 146 (24.0%) “Not classified”. Median progression-free survival was shorter among those reclassified as R2-ISS stage II (HR 1.52, 95% CI 0.979–2.358), stage III (HR 2.59, 95% CI 1.709–3.923), and stage IV (HR 3.51, 95% CI 2.124–5.784) versus stage I. Adding isatuximab led to longer progression-free survival versus doublet therapy (adjusted HR 0.544 [95% CI 0.436–0.680]), with a consistent treatment effect observed across all R2-ISS stages. This is the first study to validate the R2-ISS with novel agents, including anti-CD38 monoclonal antibodies, and to show that R2-ISS, as a prognostic scoring system, can be applied to patients with relapsed/refractory multiple myeloma.

## Introduction

In 2015, the International Staging System (ISS; which considered β2-microglobulin and serum albumin levels) [1] underwent revision to the R-ISS [2] to account for the prognostic impact of serum lactate dehydrogenase levels and certain high-risk chromosomal abnormalities [del(17p), t(4;14), and t(14;16)] among patients with newly diagnosed multiple myeloma. Though the R-ISS provided a valuable staging system for patients with newly diagnosed [2, 3] or relapsed and refractory multiple myeloma [3], significant heterogeneity was noted among the large population of patients classified as R-ISS stage II [4].

Recently, the R-ISS was further revised (R2-ISS) [5]. The R2-ISS includes gain or amplification of 1q21 (1q21+ ) in the scoring schema. D’Agostino et al. found the presence of 1q21+ to be a significant predictor of both progression-free survival (PFS) and overall survival (OS) among patients with newly diagnosed multiple myeloma [5], which was in line with previous identification of 1q21+ as a poor prognostic indicator [6, 7]. Unlike the R-ISS, t(14;16) was not included in the scoring system for R2-ISS, as it was found to be a significant risk factor for OS but not PFS [5]. In addition, the designation of t(14;16) as a rare but important independent marker of high-risk disease had already been called into question [8, 9]. The R2-ISS also accounted for the additive prognostic significance of having multiple high-risk cytogenetic abnormalities present [5, 6, 10]. Ultimately improving the ability to discriminate between the large number of patients that the R-ISS classified as “intermediate-risk” by splitting this group into low-intermediate (R2-ISS stage II) and intermediate-high (R2-ISS stage III) [5]. While there are risk factors that are prognostic for poorer PFS and OS, some risk factors have a greater influence on these outcomes than others.

As with previous staging systems, the R2-ISS was originally validated using data from clinical trials of patients with newly diagnosed multiple myeloma [5]. In their publication of the R2-ISS [5], D’Agostino et al. suggested that its value as a prognostic scoring system should be explored among patients with relapsed or relapsed and refractory multiple myeloma and among patients treated with new combinations (e.g., carfilzomib-containing regimens and triplet regimens that include monoclonal antibodies).

Isatuximab is an IgG1 monoclonal antibody that targets a unique epitope of CD38, a transmembrane glycoprotein uniformly expressed on myeloma cells [11–13]. Isatuximab achieves myeloma cell killing via multiple mechanisms, including antibody-directed cellular cytotoxicity, antibody-dependent cellular phagocytosis, complement-dependent cytotoxicity, direct apoptosis, direct activation of natural killer cells, and inhibition of CD38 ectoenzyme activity [11–14]. Based on the primary analysis of the phase 3 ICARIA-MM trial [15], isatuximab, in combination with pomalidomide and dexamethasone, is approved in several countries for patients with relapsed or refractory multiple myeloma following 2 or more prior therapies, including lenalidomide and a proteasome inhibitor [16–18]. An updated analysis of OS from ICARIA-MM has since been published [19]. Based on a preplanned interim analysis of the phase 3 IKEMA trial [20], isatuximab, in combination with carfilzomib and dexamethasone, is also approved in various countries for patients with relapsed or refractory multiple myeloma who have received at least 1 prior therapy [16, 17]. A prespecified follow-up analysis of the IKEMA study, including the final analysis of PFS, has recently been published [21]. The OS analysis from IKEMA was published recently [22].

The primary aim of our study was to validate the prognostic value of the R2-ISS staging system among patients with relapsed or refractory multiple myeloma using large datasets from the ICARIA-MM and IKEMA trials. We also aimed to evaluate the impact of early relapse on R2-ISS staging and to examine the benefit of isatuximab-based triplet therapy (isatuximab–pomalidomide–dexamethasone [Isa-Pd] or isatuximab–carfilzomib–dexamethasone [Isa-Kd]) versus that of doublet therapy (Pd or Kd) among participants of ICARIA-MM and IKEMA, by R2-ISS stage.

## Methods

### Study design and participants

This was a retrospective analysis of patients with relapsed or refractory multiple myeloma who were enrolled and randomized in the ICARIA-MM (between Jan 10, 2017, and Feb 2, 2018) and IKEMA (between Nov 15, 2017, and March 21, 2019) trials, as previously described (Supplemental Fig. S1, Supplementary Fig. S2) [15, 19–21, 23, 24]. Each trial was previously approved by the relevant ethics committee in each study site. All patients provided written informed consent in accordance with the Declaration of Helsinki. All methods were performed in accordance with the relevant guidelines and regulations.

Briefly, patients aged ≥18 years who had received ≥2 (ICARIA-MM) or 1–3 previous lines of therapy (IKEMA) were eligible for the studies. Patients in ICARIA-MM were required to be refractory to lenalidomide and a proteasome inhibitor, given alone or in combination. Patients were excluded from both trials if they had anti-CD38–refractory disease or had previously received pomalidomide (ICARIA-MM) or carfilzomib (IKEMA). In ICARIA-MM, patients were randomized 1:1 to Isa-Pd or Pd. In IKEMA, patients were randomized 3:2 to Isa-Kd or Kd.

Patients in both ICARIA-MM and IKEMA were assessed for ISS disease stage at study entry. During screening (for ICARIA-MM) or at baseline (for IKEMA), lactate dehydrogenase levels were assessed by local laboratories, with upper limits of normal defined by the individual laboratories. The presence of del(17p), t(4;14), and 1q21+ was assessed by central laboratory fluorescence in-situ hybridization testing after immunomagnetic isolation of CD138+ plasma cells from baseline bone marrow aspirate. Cytogenetics were assessed during screening for both ICARIA-MM and IKEMA, with 1 exception: 1q21+ was assessed retrospectively for ICARIA-MM participants after study completion using remaining CD138+ cells. Cutoffs used for positivity were 50% for del(17p) and 30% for t(4;14) and 1q21+.

### Procedures

Using data collected at the time of relapse or refractoriness, participants of the ICARIA-MM and IKEMA studies were reclassified into R2-ISS stage according to the protocol outlined by D’Agostino et al. [5]. A score value (in brackets) was assigned to available individual prognostic risk factors considered for R2-ISS staging: ISS stage II [1.0]; ISS stage III [1.5]; lactate dehydrogenase above the upper limit of normal [1.0]; presence of del(17p) [1.0]; presence of t(4;14) [1.0]; and presence of 1q21+ [0.5]. The sum of risk factor values was used to classify patients according to R2-ISS stage, as follows: 0, stage I; 0.5 to 1.0, stage II; 1.5 to 2.5, stage III, and ≥3.0, stage IV.

To minimize the number of patients deemed not classifiable, an allowance was made for missing data when the sum of available risk factors reached a certain threshold. If patients had 1 missing risk factor, and the missing risk factor was not ISS stage, and the total score of existing non-missing risk factors was 1.5, then R2-ISS was classified as stage III irrespective of the score value assigned to the missing risk factor. If the total score of non-missing risk factors was ≥3.0, patients were designated as R2-ISS stage IV, irrespective of the number of missing risk factors. Patients who did not meet criteria for allocation into R2-ISS stage I, II, III, or IV, according to the above definitions, were designated “Not classified”.

For the subgroup analysis of patients with early relapse, “early relapse” was defined as previously described [25–27]: relapse <12 months from initiation of the most recent line of therapy (for patients with ≥2 prior lines of therapy), relapse <18 months for patients with 1 prior line of therapy, or relapse <12 months from the time of autologous stem-cell transplant. Primary refractory patients could not be considered as having early relapse.

### Statistical analysis

Continuous data were summarized using the number of available data, median, and IQR. Categorical and ordinal data were summarized using the number and percentage of patients. The PFS analysis included data from ICARIA-MM (data cutoff Oct 11, 2018) and IKEMA (data cutoff Jan 14, 2022). The exploratory analysis of OS included data from ICARIA-MM (data cutoff Jan 27, 2022) and IKEMA (data cutoff Feb 7, 2023).

PFS was defined as the time from the date of randomization to the date of first documentation of progressive disease (as determined by an independent response committee [IRC]) or the date of death from any cause, whichever came first. Patients were assessed for progression by the IRC using M-protein quantification from central laboratory and central review of imaging. OS was defined as the time from the date of randomization to the date of death from any cause.

Pooled data from ICARIA-MM and IKEMA were used to construct validation curves showing survival outcomes for patients grouped into each R2-ISS stage. Survival endpoints (25% quantile, median, and 75% quantile with corresponding 95% confidence intervals [CIs]) were analyzed using the Kaplan-Meier method. Log-rank tests and Cox regression models, stratified and adjusted by treatment, respectively, were used to generate 1-sided *p*-values (at a 2.5% significance level) and hazard ratios (HRs) to compare outcomes between patients grouped into R2-ISS stage II, III, or IV versus those classified as stage I. Survival endpoints were also analyzed according to the presence or absence of individual risk factors considered for R2-ISS staging.

To examine outcomes by Isa-based triplet versus doublet (pooled data from ICARIA-MM and IKEMA), Cox regression models (stratified by R2-ISS stage) were used to assess PFS and OS for all patients (adjusted by R2-ISS stage) and by individual subgroups of patients according to R2-ISS stage. Separate survival analyses were also conducted by individual study, and outcomes were analyzed using interaction tests from the Cox proportional-hazards model with terms for the factor, treatment effect, and the treatment-by-factor interaction.

All statistical analyses were conducted using SAS version 9.4.

## Results

**Fig. 1:**
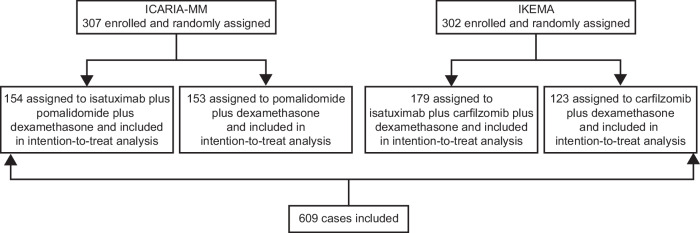
Patients from the ICARIA-MM and IKEMA trials included in the present analysis.

Data from 307 participants in the ICARIA-MM trial and 302 participants in the IKEMA trial (Fig. 1) were analyzed. A summary of selected baseline patient characteristics from each study, as previously reported [15, 20], is shown in Table 1. Classification of each study’s participants by risk factors considered for R2-ISS staging, and by re-allocation into R2-ISS stage, is shown in Table 2. Overall, more patients in IKEMA (53.0% vs. 36.8% in ICARIA-MM) were ISS stage I at study entry and fewer were stage III at study entry (15.2%] vs. 25.1%). Both patterns are in line with a greater proportion of patients from IKEMA being reclassified as R2-ISS stage I (15.9% vs. 6.5%).

**Table 1:** Selected baseline demographics of randomized populations from ICARIA-MM and IKEMA.

	ICARIA-MM		IKEMA	
	Isatuximab plus pomalidomide plus dexamethasone (*n* = 154)	Pomalidomide plus dexamethasone (*n* = 153)	Isatuximab plus carfilzomib plus dexamethasone (*n* = 179)	Carfilzomib plus dexamethasone (*n* = 123)
Age (years)				
Median	68 (60–74)	66 (59–71)	65 (55–70)	63 (57–70)
<65	54 (35%)	70 (46%)	88 (49%)	66 (54%)
≥65 to <75	68 (44%)	54 (35%)	74 (41%)	47 (38%)
≥75	32 (21%)	29 (19%)	17 (9%)	10 (8%)
Gender				
Female	65 (42%)	83 (54%)	78 (44%)	55 (45%)
Male	89 (58%)	70 (46%)	101 (56%)	68 (55%)
eGFR^a^ <60 mL/min per 1.73 m^2^	55/142 (39%)	49/145 (34%)	43/165 (26%)	18/111 (16%)
Previous autologous stem-cell transplant	83 (54%)	90 (59%)	116 (65%)	69 (56%)
Time from initial diagnosis of multiple myeloma to randomization, years	4.5 (2.6–7.2)	4.1 (2.9–7.0)	3.2 (2.0–5.5)	3.3 (2.1–5.8)
Number of previous lines of therapy	3 (2–4)	3 (2–4)	2 (1–2)	2 (1–3)
Previous therapy				
Alkylating agents	139 (90%)	148 (97%)	169 (94%)	101 (82%)
Proteasome inhibitors	154 (100%)	153 (100%)	166 (93%)	105 (85%)
Immunomodulatory agents	154 (100%)	153 (100%)	136 (76%)	100 (81%)
Monoclonal antibodies	0	0	5 (3%)	1 (1%)
Refractory to treatment				
Last regimen	150 (97%)	151 (99%)	89 (50%)	73 (59%)
Immunomodulatory agent	147 (95%)	144 (94%)	78 (44%)	58 (47%)
Proteasome inhibitor	118 (77%)	115 (75%)	56 (31%)	44 (36%)
Lenalidomide	144 (94%)	140 (92%)	57 (32%)	42 (34%)
Lenalidomide in last previous regimen	93 (60%)	88 (58%)	36 (20%)	31 (25%)

Data are *n* (%), *n/N* (%), or median (IQR) unless otherwise indicated.

^a^Calculated using the MDRD equation in patients with race reported in case report form. For ICARIA-MM, patients with creatinine clearance <30 mL/min were excluded; for IKEMA, patients with eGFR <15 mL/min per 1.73 m^2^ were excluded. *eGFR* estimated glomerular filtration rate, *MDRD* modification of diet in renal disease, *IQR* interquartile range.

**Table 2:** R2-ISS stage and risk factors considered for R2-ISS staging (patients from ICARIA and IKEMA).

	ICARIA			IKEMA		
	Isatuximab plus pomalidomide plus dexamethasone (*n* = 154)	Pomalidomide plus dexamethasone (*n* = 153)	All (*N* = 307)	Isatuximab plus carfilzomib plus dexamethasone (*n* = 179)	Carfilzomib plus dexamethasone (*n* = 123)	All (*N* = 302)
ISS stage at study entry						
Stage I	62 (40.3%)	51 (33.3%)	113 (36.8%)	89 (49.7%)	71 (57.7%)	160 (53.0%)
Stage II	55 (35.7%)	56 (36.6%)	111 (36.2%)	63 (35.2%)	31 (25.2%)	94 (31.1%)
Stage III	34 (22.1%)	43 (28.1%)	77 (25.1%)	26 (14.5%)	20 (16.3%)	46 (15.2%)
Unknown	3 (1.9%)	3 (2.0%)	6 (2.0%)	1 (0.6%)	1 (0.8%)	2 (0.7%)
del(17p)^a^						
Present	14 (9.1%)	23 (15.0%)	37 (12.1%)	18 (10.1%)	16 (13.0%)	34 (11.3%)
Absent	118 (76.6%)	95 (62.1%)	213 (69.4%)	143 (79.9%)	96 (78.0%)	239 (79.1%)
Unknown or missing	22 (14.3%)	35 (22.9%)	57 (18.6%)	18 (10.1%)	11 (8.9%)	29 (9.6%)
Serum lactate dehydrogenase^b^						
≤Upper limit of normal	106 (68.8%)	102 (66.7%)	208 (67.8%)	137 (76.5%)	97 (79.5%)	234 (77.7%)
>Upper limit of normal	48 (31.2%)	51 (33.3%)	99 (32.2%)	42 (23.5%)	25 (20.5%)	67 (22.3%)
Missing	0	0	0	0	1 (<0.1%)	1 (<0.1%)
t(4;14)^a^						
Present	12 (7.8%)	14 (9.2%)	26 (8.5%)	22 (12.3%)	20 (16.3%)	42 (13.9%)
Absent	119 (77.3%)	101 (66.0%)	220 (71.7%)	137 (76.5%)	89 (72.4%)	226 (74.8%)
Unknown or missing	23 (14.9%)	38 (24.8%)	61 (19.9%)	20 (11.2%)	14 (11.4%)	34 (11.3%)
1q21+^c^						
Present	76 (49.4%)	52 (34.0%)	128 (41.7%)	75 (41.9%)	52 (42.3%)	127 (42.1%)
Absent	38 (24.7%)	46 (30.1%)	84 (27.4%)	84 (46.9%)	55 (44.7%)	139 (46.0%)
Unknown or missing	40 (26.0%)	55 (35.9%)	95 (30.9%)	20 (11.2%)	16 (13.0%)	36 (11.9%)
R2-ISS stage						
Stage I	11 (7.1%)	9 (5.9%)	20 (6.5%)	31 (17.3%)	17 (13.8%)	48 (15.9%)
Stage II	27 (17.5%)	24 (15.7%)	51 (16.6%)	47 (26.3%)	38 (30.9%)	85 (28.1%)
Stage III	52 (33.8%)	47 (30.7%)	99 (32.2%)	68 (38.0%)	37 (30.1%)	105 (34.8%)
Stage IV	16 (10.4%)	18 (11.8%)	34 (11.1%)	11 (6.1%)	10 (8.1%)	21 (7.0%)
Not classified	48 (31.2%)	55 (35.9%)	103 (33.6%)	22 (12.3%)	21 (17.1%)	43 (14.2%)

Data are *n* (%). ^a^del(17p) and t(4;14) were assessed during screening for ICARIA-MM and IKEMA by a central laboratory with a cutoff of 50% and 30%, respectively. ^b^Lactate dehydrogenase assessment at baseline for IKEMA: isatuximab–carfilzomib–dexamethasone (*n* = 179); carfilzomib–dexamethasone (*n* = 122); all (*N* = 301). ^c^1q21+ (cutoff of 30%) was assessed by a central laboratory prospectively during screening for IKEMA and retrospectively for ICARIA-MM. *ISS* International Staging System, *R2-ISS* Second Revision of the International Staging System.

Regarding other factors considered for re-allocation, the greatest discrepancy between IKEMA and ICARIA-MM participants was in the volume of 1q21+ data available for analysis: 36 (11.9%) of 302 patients in IKEMA versus 95 (30.9%) of 307 patients in ICARIA-MM had missing 1q21+ data. This was attributed to the retrospective nature of 1q21+ assessment in ICARIA-MM (due to lack of leftover material and patient consent withdrawal) compared with the prospective analysis in IKEMA and is in line with a lower proportion of patients being designated as R2-ISS “Not classified” in IKEMA versus ICARIA-MM (43 [14.2%] of 302 patients vs. 103 [33.6%] of 307 patients, respectively).

Of the 609 patients from the pooled ICARIA-MM and IKEMA study populations, 68 (11.2%) were reclassified as R2-ISS stage I, 136 (22.3%) as R2-ISS stage II, 204 (33.5%) as R2-ISS stage III, 55 (9.0%) as stage IV, and 146 (24.0%) were “Not classified”. The distribution of single risk factors present among pooled patients within each R2-ISS stage is shown in Table 3.

**Table 3:** Risk factors for patients included in R2-ISS stages (pooled data from ICARIA-MM and IKEMA).

	R2-ISS stage					
	Stage I (*n* = 68)	Stage II (*n* = 136)	Stage III (*n* = 204)	Stage IV (*n* = 55)	Not classified (*n* = 146)	All (*n* = 609)
No risk factors present	68 (100%)	0	0	0	0	68 (11.2%)
ISS stage II at study entry	0	48 (35.3%)	89 (43.6%)	15 (27.3%)	53 (36.3%)	205 (33.7%)
ISS stage III at study entry	0	0	62 (30.4%)	39 (70.9%)	22 (15.1%)	123 (20.2%)
Lactate dehydrogenase >upper limit of normal	0	19 (14.0%)	58 (28.4%)	47 (85.5%)	42 (28.8%)	166 (27.3%)
del(17p)^a^ present	0	10 (7.4%)	25 (12.3%)	27 (49.1%)	9 (6.2%)	71 (11.7%)
t(4;14)^a^ present	0	6 (4.4%)	42 (20.6%)	18 (32.7%)	2 (1.4%)	68 (11.2%)
1q21+^b^ present	0	53 (39.0%)	142 (69.6%)	47 (85.5%)	13 (8.9%)	255 (41.9%)

Data are *n* (%). ^a^del(17p) and t(4;14) were assessed during screening for ICARIA-MM and IKEMA by a central laboratory with a cutoff of 50% and 30%, respectively. ^b^1q21+ (cutoff of 30%) was assessed by a central laboratory during screening for IKEMA and retrospectively for ICARIA-MM. *R2-ISS* Second Revision of the International Staging System, *ISS* International Staging System.

**Fig. 2:**
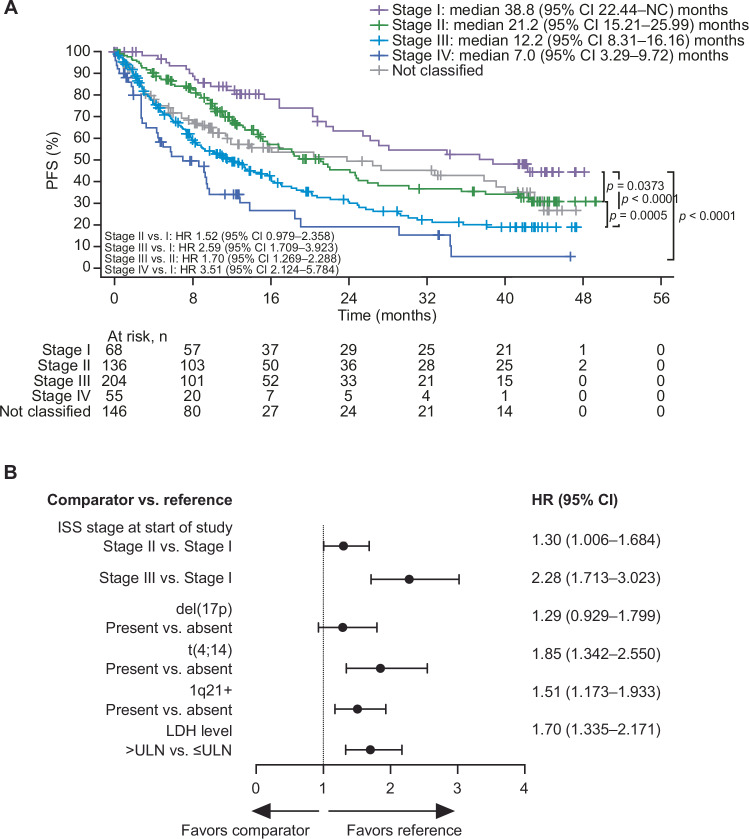
Pooled data from ICARIA-MM and IKEMA. **A** validation curves showing progression-free survival by R2-ISS stage. **B** hazard ratios of progression-free survival by subgroups with individual risk factors. CI confidence interval, HR hazard ratio, ISS International Staging System, LDH lactate dehydrogenase, NC not calculable, PFS progression-free survival, R2-ISS Second Revision of the International Staging System, ULN upper limit of normal.

Validation curves for PFS are shown in Fig. 2A; number of progression events, number of patients censored, and Kaplan-Meier estimates for quantiles and medians are provided in Supplementary Table S1. After a median follow-up duration of 11.6 months (IQR 10.1–13.9) in ICARIA-MM and 44.0 months (IQR 42.3–45.4) in IKEMA, median PFS was shorter among pooled patients reclassified as R2-ISS stage II compared with stage I (Fig. 2A). Median PFS was also shorter among pooled patients reclassified as R2-ISS stage III compared with stage II. The median PFS decreased with increasing R2-ISS stage. The presence of individual risk factors considered for R2-ISS staging (compared with their absence) were similarly associated with shorter PFS (Fig. 2B).

**Fig. 3:**
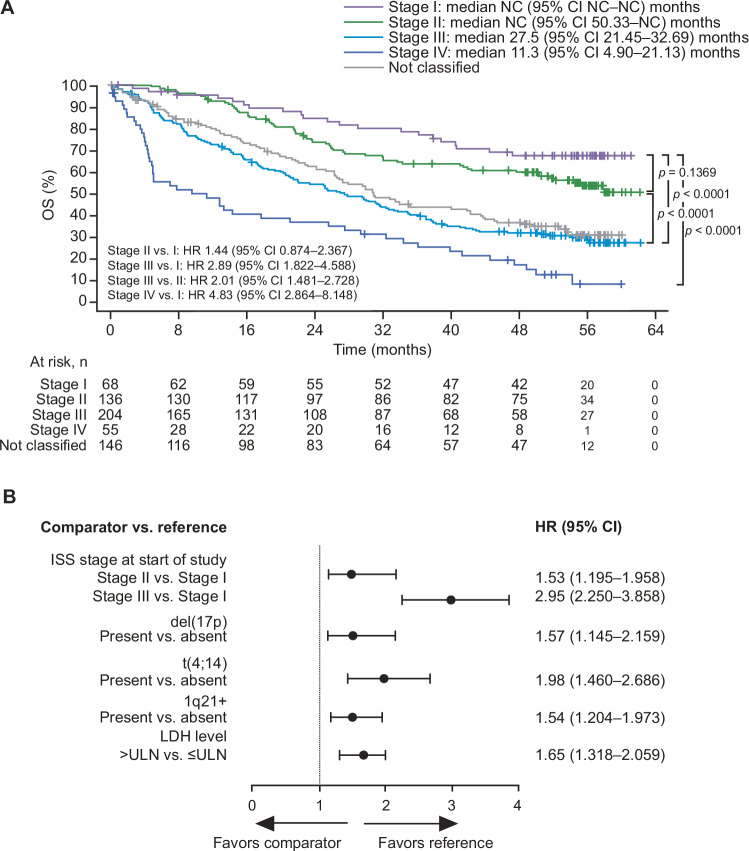
Pooled data from ICARIA-MM and IKEMA. **A** validation curves showing overall survival by R2-ISS stage. **B** hazard ratios of overall survival by subgroups with individual risk factors. CI confidence interval, HR hazard ratio, ISS International Staging System, LDH lactate dehydrogenase, NC not calculable, OS overall survival, R2-ISS Second Revision of the International Staging System, ULN upper limit of normal.

Validation curves for OS are shown in Fig. 3A; number of deaths, number of patients censored, and Kaplan-Meier estimates for quantiles and medians are provided in Supplementary Table S2. After a median follow-up duration of 52.4 months (IQR 50.66–54.80) in ICARIA-MM and 56.61 months (IQR 54.90–58.02) in IKEMA, median OS was shorter among pooled patients reclassified as R2-ISS stage II, stage III, and stage IV compared with stage I (Fig. 3A). OS was also shorter among pooled patients reclassified as R2-ISS stage III compared with stage II. Median OS was not reached for R2-ISS stage I or stage II and was 27.5 months (95% CI 21.45–32.69) and 11.3 months (95% CI 4.90–21.13) for stages III and IV, respectively. There was a clear separation of the curves observed despite stage I and II medians not being reached. The presence of individual R2-ISS risk factors (compared with their absence) was similarly associated with shorter OS (Fig. 3B).

**Fig. 4:**
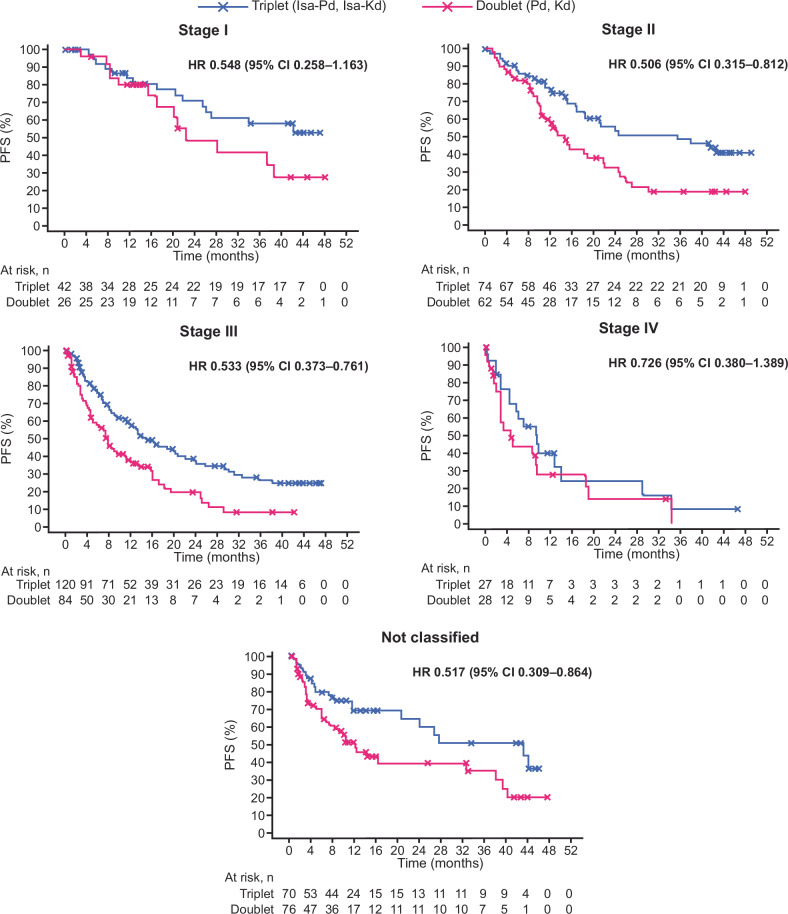
Progression-free survival (Isa-based triplet vs. doublet), by R2-ISS stage (pooled data from ICARIA-MM and IKEMA based on disease assessment by the independent response committees). CI confidence interval, HR hazard ratio, Isa-Kd isatuximab–carfilzomib–dexamethasone, Isa-Pd isatuximab–pomalidomide–dexamethasone, PFS progression-free survival, R2-ISS Second Revision of the International Staging System.

We also analyzed PFS and OS, by isatuximab-based triplet therapy versus doublet therapy, overall and by subgroups defined by R2-ISS stage. In the overall pooled population, adding isatuximab to Pd or Kd led to longer PFS compared with receiving doublet therapy (median of 23.89 months [95% CI 18.431–29.207] vs. 11.83 months [95% CI 9.528–15.376], respectively; adjusted HR [aHR] 0.544 [95% CI 0.436–0.680]). A consistent treatment effect was observed across all R2-ISS stages, including patients who were re-allocated into the R2-ISS “Not classified” category (Fig. 4). Patients who received isatuximab-based triplet therapy also had longer median OS compared with those who received doublet therapy (42.38 months [95% CI 33.676–52.698] vs. 30.49 months [95% CI 26.316–36.238], respectively; aHR 0.772 [95% CI 0.626–0.951]). Again, a consistent treatment effect was observed across all R2-ISS stages (Supplementary Fig. S3). Kaplan-Meier analyses for PFS and OS by R2-ISS stage for the individual trial overall populations are shown in Supplemental Figs. S4-S7.

For the subgroup analysis of patients with early relapse, classification of study participants with early relapse by risk factors considered for R2-ISS staging, and by re-allocation into R2-ISS stage, is shown in Table 4. Of the 294 pooled patients with early relapse, 21 (7.1%) were reclassified as R2-ISS stage I, 51 (17.3%) as R2-ISS stage II, 114 (38.8%) as R2-ISS stage III, 35 (11.9%) as stage IV, and 73 (24.8%) were “Not classified”. Compared with the whole population, more patients with early relapse were classified as R2-ISS stages III and IV (149 of 294 patients [50.7%] vs. 259 of 609 patients [42.5%]) and fewer were classified as R2-ISS stages I and II (72 of 294 patients [24.5%] vs. 204 of 609 patients [33.5%]). Consistent with results from the whole population, PFS was shorter among early relapse patients reclassified as R2-ISS stage II (HR 3.41, 95% CI 1.408–8.260), stage III (HR 5.24, 95% CI 2.261–12.125), and stage IV (HR 7.33, 95% CI 2.942–18.242) compared with stage I. Median PFS for early relapse patients was generally shorter than in the overall population and consistently decreased with increasing R2-ISS stage: stage I, not reached (95% CI 16.99–not reached); stage II, 16.9 months (95% CI 12.06–24.18); stage III, 7.9 months (95% CI 5.75–11.53); and stage IV, 4.6 months (95% CI 2.83–9.23) (Supplementary Table S3). Adding isatuximab to Pd or Kd led to improved PFS compared with doublet therapy among early relapsers (median of 14.78 months [95% CI 9.232–24.181] vs. 8.31 months [95% CI 5.585–12.057], respectively; aHR 0.624 [95% CI 0.459–0.848]) (Supplementary Table S4; Supplementary Fig. S8). A consistent treatment effect was observed across patients re-allocated into R2-ISS stages, particularly stages II–IV and the R2-ISS “Not classified” category (Supplementary Table S4). Adding isatuximab to Pd or Kd led to improved OS compared with doublet therapy among early relapsers (median of 31.11 months [95% CI 23.031–37.651] vs. 22.70 months [95% CI 14.982–29.733], respectively; aHR 0.775 [95% CI 0.590–1.018]) (Supplementary Table S5; Supplementary Fig. S9). A consistent treatment effect was observed across patients re-allocated into R2-ISS stages, except for stage II (Supplementary Table S5).

**Table 4:** Summary of R2-ISS stage and risk factors considered for R2-ISS staging (patients with early relapse from ICARIA and IKEMA).

	ICARIA			IKEMA		
	Isatuximab plus pomalidomide plus dexamethasone (*n* = 93)	Pomalidomide plus dexamethasone (*n* = 94)	All (*N* = 187)	Isatuximab plus carfilzomib plus dexamethasone (*n* = 61)	Carfilzomib plus dexamethasone (*n* = 46)	All (*N* = 107)
ISS stage at study entry						
Stage I	36 (38.7%)	24 (25.5%)	60 (32.1%)	19 (31.1%)	25 (54.3%)	44 (41.1%)
Stage II	35 (37.6%)	40 (42.6%)	75 (40.1%)	28 (45.9%)	12 (26.1%)	40 (37.4%)
Stage III	22 (23.7%)	28 (29.8%)	50 (26.7%)	14 (23.0%)	9 (19.6%)	23 (21.5%)
Unknown	0	2 (2.1%)	2 (1.1%)	0	0	0
del(17p)^a^						
Present	8 (8.6%)	13 (13.8%)	21 (11.2%)	10 (16.4%)	8 (17.4%)	18 (16.8%)
Absent	70 (75.3%)	59 (62.8%)	129 (69.0%)	45 (73.8%)	36 (78.3%)	81 (75.7%)
Unknown or missing	15 (16.1%)	22 (23.4%)	37 (19.8%)	6 (9.8%)	2 (4.3%)	8 (7.5%)
Serum lactate dehydrogenase						
≤Upper limit of normal	63 (67.7%)	57 (60.6%)	120 (64.2%)	43 (70.5%)	35 (76.1%)	78 (72.9%)
>Upper limit of normal	30 (32.3%)	37 (39.4%)	67 (35.8%)	18 (29.5%)	11 (23.9%)	29 (27.1%)
Missing	0	0	0	0	0	0
t(4;14)^a^						
Present	9 (9.7%)	10 (10.6%)	19 (10.2%)	7 (11.5%)	11 (23.9%)	18 (16.8%)
Absent	71 (76.3%)	60 (63.8%)	131 (70.1%)	48 (78.7%)	33 (71.7%)	81 (75.7%)
Unknown or missing	13 (14.0%)	24 (25.5%)	37 (19.8%)	6 (9.8%)	2 (4.3%)	8 (7.5%)
1q21+^b^						
Present	48 (51.6%)	32 (34.0%)	80 (42.8%)	25 (41.0%)	26 (56.5%)	51 (47.7%)
Absent	18 (19.4%)	28 (29.8%)	46 (24.6%)	29 (47.5%)	17 (37.0%)	46 (43.0%)
Unknown or missing	27 (29.0%)	34 (36.2%)	61 (32.6%)	7 (11.5%)	3 (6.5%)	10 (9.3%)
R2-ISS stage						
Stage I	6 (6.5%)	5 (5.3%)	11 (5.9%)	5 (8.2%)	5 (10.9%)	10 (9.3%)
Stage II	12 (12.9%)	12 (12.8%)	24 (12.8%)	15 (24.6%)	12 (26.1%)	27 (25.2%)
Stage III	36 (38.7%)	30 (31.9%)	66 (35.3%)	27 (44.3%)	21 (45.7%)	48 (44.9%)
Stage IV	10 (10.8%)	14 (14.9%)	24 (12.8%)	7 (11.5%)	4 (8.7%)	11 (10.3%)
Not classified	29 (31.2%)	33 (35.1%)	62 (33.2%)	7 (11.5%)	4 (8.7%)	11 (10.3%)

Data are *n* (%). Early relapse defined as relapse <12 months from initiation of the most recent line of therapy (for patients with ≥2 prior lines of therapy), relapse <18 months for patients with 1 prior line of therapy, or relapse <12 months from autologous stem-cell transplant. Primary refractory patients could not be considered as having early relapse. ^a^del(17p) and t(4;14) were assessed during screening for ICARIA-MM and IKEMA by a central laboratory with a cutoff of 50% and 30%, respectively. ^b^1q21+ (cutoff of 30%) was assessed by a central laboratory during screening for IKEMA and retrospectively for ICARIA-MM. *ISS* International Staging System, *R2-ISS* Second Revision of the International Staging System.

## Discussion

In this study, the 5 prognostic risk factors included in the R2-ISS [5] were used to re-allocate participants of ICARIA-MM and IKEMA into R2-ISS stages and examine the association between R2-ISS stage and survival outcomes. To our knowledge, this is the first study to independently validate the prognostic value of the R2-ISS staging system in patients with relapsed or refractory multiple myeloma, using data from 2 large phase 3 studies (609 pooled patients). It is also the first study to validate the R2-ISS among clinical trial patients receiving therapy with an anti-CD38 monoclonal antibody.

Consistent with findings from the original R2-ISS validation study in newly diagnosed multiple myeloma [5], this validation among relapsed or refractory clinical trial participants showed that re-allocation of patients from ICARIA and IKEMA into R2-ISS stages was able to demonstrate 4 subgroups that showed a progressive decline in median PFS with increasing disease stage (stage I, 38.8 months; stage II, 21.2 months; stage III, 12.2 months; stage IV, 7.0 months). These PFS differences reached statistical significance for stages III and IV, when each was compared with stage I. Median PFS was 17.6 months longer for stage I versus stage II, but the difference between these groups did not reach statistical significance. Notably, this is similar to findings from the validation cohort of newly diagnosed patients analyzed by D’Agostino et al., where median PFS was 11 months longer for stage I versus stage II (39 vs. 28 months) and the difference did not reach statistical significance (HR 1.25, 95% CI 0.99–1.59; *p* = 0.061). However, in their training cohort, D’Agostino et al. did see a statistically significant difference in PFS between these groups (median 68 months for stage I vs. 45 months for stage II; HR 1.52, 95% CI 1.30–1.77; *p* <0.0001). Smaller cohort sizes and the limited number of events due to limited follow-up could have contributed to the lack of statistical significance in our study (609 pooled patients) and in the validation cohort of D’Agostino et al. (1214 evaluable patients) compared with their training cohort (2226 evaluable patients). Furthermore, if a patient is not “high risk” according to R2-ISS, other factors might interact. In D’Agostino et al., the difference between stage II and stage I in transplant-ineligible patients with newly diagnosed multiple myeloma was not statistically significant, which could be due to the interaction of other factors included in the score, such as frailty. In the current study, the same thing may have occurred in patients allocated to stage II versus I with other factors such as frailty, drug exposure, or drug refractoriness that might interact with the risk conferred by R2-ISS.

The validation curves for OS suggest a progressive decline in OS as R2-ISS stage progresses from stage I to stage IV. These OS differences were statistically significant for stages III and IV when compared with stage I; however, we were not able to discriminate well between OS among stage II versus stage I patients, likely because patients with lower disease stage would be expected to survive longer than those with more advanced disease stage at baseline. Furthermore, stage II is a new patient population in relapsing/refractory multiple myeloma that requires further research to elucidate this increased risk and its longer-term impact.

In line with findings from D’Agostino et al. [5] in newly diagnosed disease, our study among patients with relapsed or refractory multiple myeloma shows that the R2-ISS improved discrimination of the large number of patients that the R-ISS classified as “intermediate-risk” by splitting this group into R2-ISS stage II or III. This is evidenced by a more even distribution of patients among the 4 R2-ISS stages, as opposed to patients mostly being classified as R-ISS stage II. Additionally, improved discrimination was observed in terms of differences in PFS and OS between patients reclassified as R2-ISS stages II and III, with significantly worse survival reported in stage III patients.

In line with the primary and updated analyses of the ICARIA-MM [15, 19] and IKEMA [20, 21] trials, benefit of isatuximab-based triplet therapy (Isa-Pd or Isa-Kd) over doublet therapy (Pd or Kd) was confirmed for all patients upon reclassification according to R2-ISS stage at study entry. This analysis helps to put clinical trial data into current context as updates/improvements are made to staging strategies. Notably, the R2-ISS was originally validated using data from a cohort of patients with newly diagnosed multiple myeloma who received first-line therapy with either immunomodulatory drug-based therapy (89%) or both an immunomodulatory drug and proteasome inhibitor (excluding carfilzomib, 11%) [5]. Though performed in relapsed or refractory rather than newly diagnosed patients, our study suggests that the R2-ISS, when applied at the time of relapse or refractoriness, holds its prognostic value when patients are treated with novel agents, including isatuximab and carfilzomib.

Further analysis of our data showed that, when compared to the overall pooled population from ICARIA-MM and IKEMA, patients with early relapse were more likely to be classified as R2-ISS stage III or IV and less likely to be classified as stage I or II. Re-allocation of patients with early relapses into R2-ISS stages was still able to demonstrate 4 subgroups that showed a progressive decline in median PFS with increasing disease stage. We also found a clear benefit of isatuximab-based triplet therapy over doublet therapy among pooled patients with early relapse (aHR 0.624 [95% CI 0.459–0.848]), consistent with findings among early-relapsing patients from the IKEMA trial alone (median PFS of 24.7 months with Isa-Kd vs. 17.2 months with Kd; HR 0.662 [95.4% CI 0.404–1.087]) [25]. This is also in line with outcomes among patients with early relapses enrolled in the CANDOR study (daratumumab-Kd vs. Kd in patients with relapsed or refractory multiple myeloma) [27].

The R2-ISS validation study by D’Agostino et al. [5] and the real-world validation study within a newly diagnosed multiple myeloma population by Tan et al. [28] allocated patients to R2-ISS stage who had available data for all 5 prognostic risk factors. Notably, D’Agostino et al. found that OS was similar among patients with incomplete versus complete cytogenetic data [5]. As such, to minimize the number of patients deemed not classifiable and to more accurately reflect how staging systems may be broadly applied in real-world practice, we allowed for missing data when the sum of available risk factors reached a certain threshold. We view this allowance as a strength of our study, as it is possible that some patients in our cohort would have been allocated to a higher R2-ISS stage if information on all risk factors was available. Although we found no major differences before and after this allowance for missing data in the overall associations that we showed between factors considered in the R2-ISS and survival outcomes (Supplementary Tables S6, S7), we underscore the importance of full laboratory and cytogenetic testing at multiple myeloma diagnosis, and this should be increasingly encouraged as standard practice across academic and community practices.

Other strengths of our study include its independent validation of the R2-ISS using 2 large datasets (pooled cohort of 609 patients) from recent phase 3 trials in relapsed or refractory multiple myeloma where baseline assessment of cytogenetic risk factors was performed centrally. Our study is also applicable to patients treated with new treatment combinations (e.g., carfilzomib-containing regimens and triplets that include a monoclonal antibody). In addition, we showed consistent benefit of isatuximab-based triplet therapy despite differences in the doublet backbone used (pomalidomide-based or carfilzomib-based), revealing the ability of isatuximab to complement therapies with varying mechanisms of action. Moving forward, the applicability of the R2-ISS among relapsed or refractory patients in real-world settings should be explored, as should its continued applicability across all disease stages as newer treatment strategies (e.g., monoclonal antibody-based quadruplet therapies, bi-specific antibodies, and chimeric antigen receptor T-cell therapies) emerge.

One limitation of our study is the retrospective nature of 1q21+ characterization in ICARIA-MM, which led to an increase in missing 1q21+ data due to lack of residual material and the operational challenge of withdrawal of patient consent. Though prospective cytogenetic profiling would have been desirable, retrospective collection may be more reflective of current cytogenetic testing procedures in clinical practice. The overall level of missing data for any cytogenetic abnormality is another limitation to our study; again, this may be reflective of real-world practice, especially in community-based settings. It is also important to comment on the use of the 30% threshold of 1q21+ positivity in this study, as there currently is no consensus, making comparison difficult with other studies that have used a different threshold. Notably, an update of the IMS high-risk criteria is forthcoming, which is expected to facilitate the consistency of these thresholds and subsequent comparisons. Another challenging aspect of our study was the shorter follow-up time for ICARIA-MM than IKEMA, particularly for IRC-determined PFS. Our study therefore did not include evaluation of the prognostic ability of equivalent R2-ISS scores based on only 1 risk factor versus based on 2 or more risk factors.

In conclusion, our study is the first to demonstrate that the R2-ISS holds its value as a simple prognostic algorithm for patients at the time of relapsed and/or refractory multiple myeloma and in the era of novel agents, including anti-CD38 monoclonal antibodies. Our findings highlight the benefit of isatuximab-based triplet therapy versus immunomodulatory drug-based or proteasome inhibitor-based doublet therapy across diverse populations of patients with relapsed and refractory multiple myeloma, including those with early relapse. Moving forward, the R2-ISS could be used to stratify patients in clinical trials enrolling patients with multiple myeloma who have been exposed to 1 or more prior lines of therapy. As more is learned about additional risk factors that influence clinical outcomes among relapsed or relapsed/refractory patients and available treatment choices, the additive nature of the R2-ISS may allow for further refinement within this population and so help translation of clinical trial results to real-world practice [29].

## Supplementary information

Supplementary Appendix

Video Abstract

## Data Availability

Qualified researchers can request access to patient-level data and related study documents including the clinical study report, study protocol with any amendments, blank case report forms, statistical analysis plan, and dataset specifications. Patient-level data will be anonymized, and study documents will be redacted to protect the privacy of trial participants. Further details on Sanofi’s data-sharing criteria, eligible studies, and process for requesting access are at: https://www.vivli.org.
