# Supplementary material for: Allocation and validation of the second revision of the International Staging System in the ICARIA-MM and IKEMA studies
Source: Blood Cancer J. 2024 Nov 28;14(1):209. doi: 10.1038/s41408-024-01149-w (PMC11605113; doi:10.1038/s41408-024-01149-w)
Supplement: Supplementary file 1 — Supplementary Appendix [file 41408_2024_1149_MOESM1_ESM.docx]

SUPPLEMENTARY APPENDIX

This appendix provides additional information about the manuscript: Richardson PG, Perrot A, Mikhael J, et al. Allocation and validation of the second revision of the International Staging System in the ICARIA-MM and IKEMA studies.

# Contents

[Contents 1](#_Toc175742550)

[Table S1. Progression-free survival by R2-ISS stage versus R2-ISS stage I (pooled intention-to-treat populations from ICARIA-MM and IKEMA). 5](#_Toc175742551)

[Table S2. Overall survival by R2-ISS stage versus R2-ISS stage I (pooled intention-to-treat populations from ICARIA-MM and IKEMA). 6](#_Toc175742552)

[Fig. S3 Overall survival (Isa-based triplet vs. doublet), by R2-ISS stage (pooled data from ICARIA-MM and IKEMA). *CI* confidence interval, *HR* hazard ratio, *Isa-Kd* isatuximab–carfilzomib–dexamethasone, *Isa-Pd* isatuximab–pomalidomide–dexamethasone, *Kd* carfilzomib–dexamethasone. *OS* overall survival, *Pd* pomalidomide–dexamethasone, *R2-ISS* Second Revision of the International Staging System. 7](#_Toc175742553)

[Fig. S5 Overall survival (isatuximab–pomalidomide–dexamethasone vs. pomalidomide–dexamethasone) in ICARIA-MM, by R2-ISS stage. *CI* confidence interval, *HR* hazard ratio, *Isa-Pd* isatuximab–pomalidomide–dexamethasone, *OS* overall survival, *Pd* pomalidomide–dexamethasone, *R2-ISS* Second Revision of the International Staging System. 9](#_Toc175742554)

[Fig. S7 Overall survival (isatuximab–carfilzomib–dexamethasone *vs* carfilzomib–dexamethasone) in IKEMA, by R2-ISS stage. *CI* confidence interval, *HR* hazard ratio, *Isa-Kd* isatuximab–carfilzomib–dexamethasone, *OS* overall survival, *R2-ISS* Second Revision of the International Staging System. 11](#_Toc175742555)

[Table S3. Progression-free survival by R2-ISS stage versus R2-ISS stage 1 (pooled early relapse populations from ICARIA-MM and IKEMA). 12](#_Toc175742556)

[Table S4. Progression-free survival (isatuximab-based triplet vs. doublet), by R2-ISS stage, among pooled early relapse populations from ICARIA-MM and IKEMA. 13](#_Toc175742557)

[Fig. S8 Progression-free survival (isatuximab-based triplet vs. doublet), by R2-ISS stage, among pooled early relapse populations from ICARIA-MM and IKEMA. 14](#_Toc175742558)

[Table S5. Overall survival (isatuximab-based triplet vs. doublet), by R2-ISS stage, among pooled early relapse populations from ICARIA-MM and IKEMA. 15](#_Toc175742559)

[Fig. S9 Overall survival (isatuximab-based triplet vs. doublet), by R2-ISS stage, among pooled early relapse populations from ICARIA-MM and IKEMA. 16](#_Toc175742560)

[Table S6. Progression-free survival by R2-ISS stage versus R2-ISS stage I (pooled intention-to-treat populations from ICARIA-MM and IKEMA) without allowance for missing data. 17](#_Toc175742561)

[Table S7. Overall survival by R2-ISS stage versus R2-ISS stage I (pooled intention-to-treat populations from ICARIA-MM and IKEMA) without allowance for missing data. 18](#_Toc175742562)

[REFERENCES 19](#_Toc175742563)

**Fig. S1 CONSORT diagram for ICARIA-MM** [3]. ^a^Investigator decision due to free light chain increase (n = 3), physician’s decision (suspected progression, n = 1), poor compliance to protocol (n = 1), investigator decided to switch treatment to daratumumab–pomalidomide–dexamethasone (n = 1), investigator kept the same isatuximab–pomalidomide–dexamethasone combination off protocol, as the product is available commercially (n = 1); patient decision to withdraw (n = 6). ^b^More than 8 weeks passed between last contact date and analysis cutoff date. ^c^Physician decision to withdraw patient (n = 1); patient decision to withdraw (n = 6). *ITT* intention-to-treat.


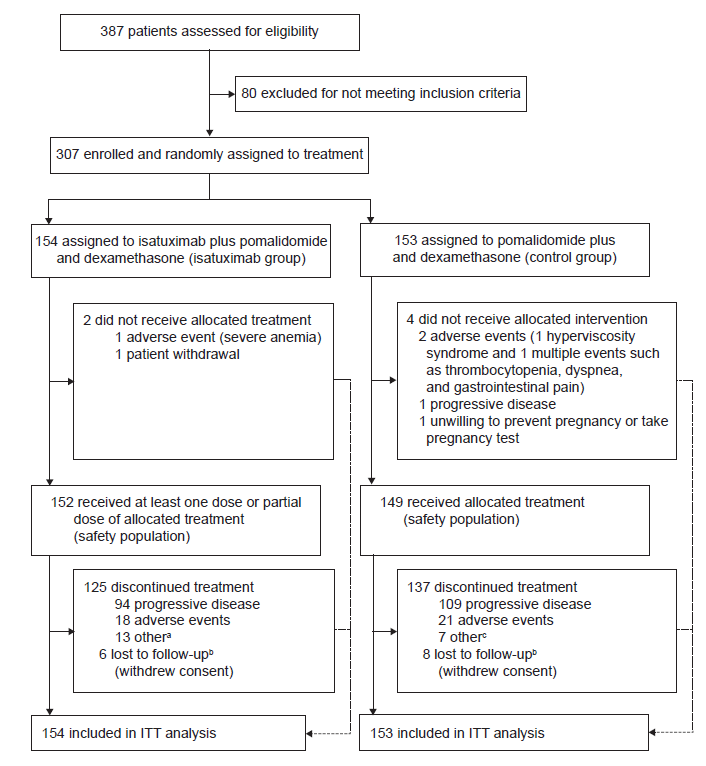


**Fig. S2** **CONSORT diagram for IKEMA** [4]. *Isa-Kd* isatuximab–carfilzomib–dexamethasone. *Kd* carfilzomib–dexamethasone. *ITT* intention-to-treat.


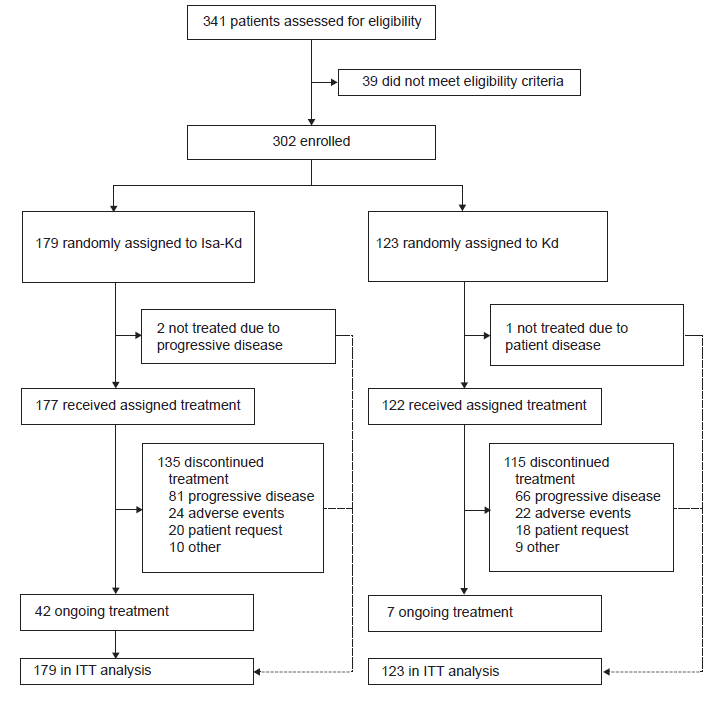


# Table S1. Progression-free survival by R2-ISS stage versus R2-ISS stage I (pooled intention-to-treat populations from ICARIA-MM and IKEMA).

|  | **R2-ISS stage** | | | |
| --- | --- | --- | --- | --- |
|  | **Stage I**  **(n = 68)** | **Stage II (n = 136)** | **Stage III (n = 204)** | **Stage IV (n = 55)** |
| Number (%) of events | 28 (41.2) | 71 (52.2) | 126 (61.8) | 37 (67.3) |
| Number (%) of patients censored | 40 (58.8) | 65 (47.8) | 78 (38.2) | 18 (32.7) |
| Kaplan-Meier estimate in months |  |  |  |  |
| 25% quantile (95% CI) (months) | 17.0 (8.57–22.44) | 10.3 (7.85–12.25) | 4.5 (3.09–6.01) | 2.8 (1.45–4.44) |
| Median survival (95% CI) (months) | 38.8 (22.44–NC) | 21.2 (15.21–25.99) | 12.2 (8.31–16.16) | 7.0 (3.29–9.72) |
| 75% quantile (95% CI) (months) | NC (NC–NC) | NC (38.11–NC) | 29.4 (20.80–NC) | 18.5 (9.43–34.40) |
| Stratified log-rank test *p*-value |  |  |  |  |
| vs. R2-ISS stage I |  | 0.037 | <0.0001 | <0.0001 |
| Adjusted^a^ hazard ratio (95% CI) |  |  |  |  |
| vs. R2-ISS stage I |  | 1.52 (0.979–2.358) | 2.59 (1.709–3.923) | 3.51 (2.124–5.784) |

^a^Adjusted by treatment. *CI* confidence interval, *NC* not calculable, *R2-ISS* Second Revision of the International Staging System.

# Table S2. Overall survival by R2-ISS stage versus R2-ISS stage I (pooled intention-to-treat populations from ICARIA-MM and IKEMA).

|  | **R2-ISS stage** | | | |
| --- | --- | --- | --- | --- |
|  | **Stage I**  **(N = 68)** | **Stage II (N = 136)** | **Stage III (N = 204)** | **Stage IV (N = 55)** |
| Number (%) of events | 21 (30.9) | 60 (44.1) | 140 (68.6) | 47 (85.5) |
| Number (%) of patients censored | 47 (69.1) | 76 (55.9) | 64 (31.4) | 8 (14.5) |
| Kaplan-Meier estimate in months |  |  |  |  |
| 25% quantile (95% CI) (months) | 38.8 (22.67–NC) | 23.7 (18.92–29.93) | 10.7 (8.44–14.42) | 4.0 (1.94–4.90) |
| Median survival (95% CI) (months) | NC (NC–NC) | NC (50.33–NC) | 27.5 (21.45–32.69) | 11.3 (4.90–21.13) |
| 75% quantile (95% CI) (months) | NC (NC–NC) | NC (NC–NC) | NC (51.42–NC) | 39.9 (21.13–49.94) |
| Stratified log-rank test *p*-value |  |  |  |  |
| vs. R2-ISS stage I |  | 0.1369 | <0.0001 | <0.0001 |
| Adjusted^a^ hazard ratio (95% CI) |  |  |  |  |
| vs. R2-ISS stage I |  | 1.44 (0.874–2.367) | 2.89 (1.822–4.588) | 4.83 (2.864–8.148) |

^a^Adjusted by treatment. *CI* confidence interval, *NC* not calculable, *R2-ISS* Second Revision of the International Staging System.

# Fig. S3 Overall survival (Isa-based triplet vs. doublet), by R2-ISS stage (pooled data from ICARIA-MM and IKEMA). *CI* confidence interval, *HR* hazard ratio, *Isa-Kd* isatuximab–carfilzomib–dexamethasone, *Isa-Pd* isatuximab–pomalidomide–dexamethasone, *Kd* carfilzomib–dexamethasone. *OS* overall survival, *Pd* pomalidomide–dexamethasone, *R2-ISS* Second Revision of the International Staging System.


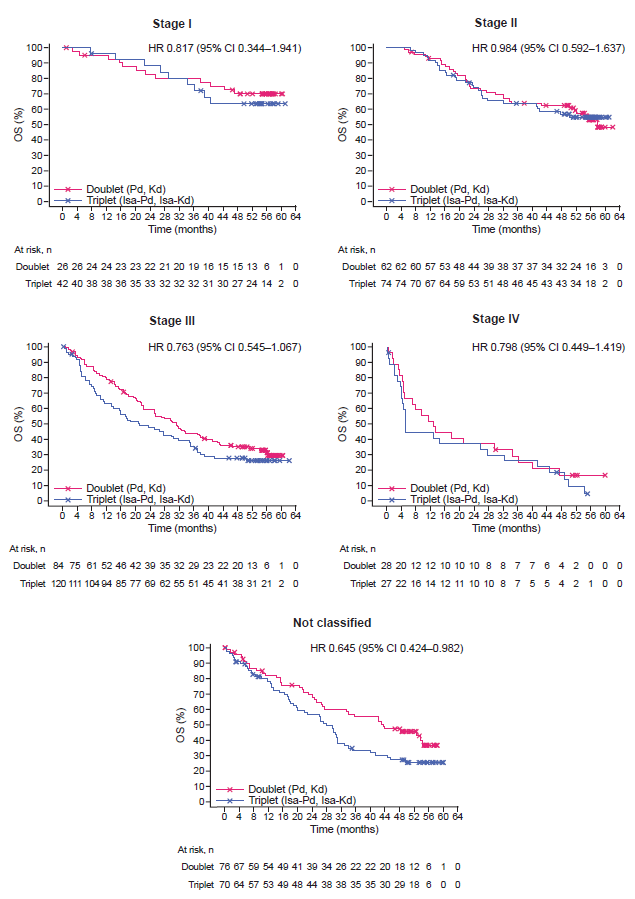


**Fig. S4 Progression-free survival (isatuximab–pomalidomide–dexamethasone *vs* pomalidomide–dexamethasone) in ICARIA-MM, by R2-ISS stage, based on disease assessment by the independent response committee.** *CI* confidence interval, *HR* hazard ratio, *Isa-Pd* isatuximab–pomalidomide–dexamethasone, *Pd* pomalidomide–dexamethasone, *PFS* progression-free survival, *R2-ISS* Second Revision of the International Staging System.


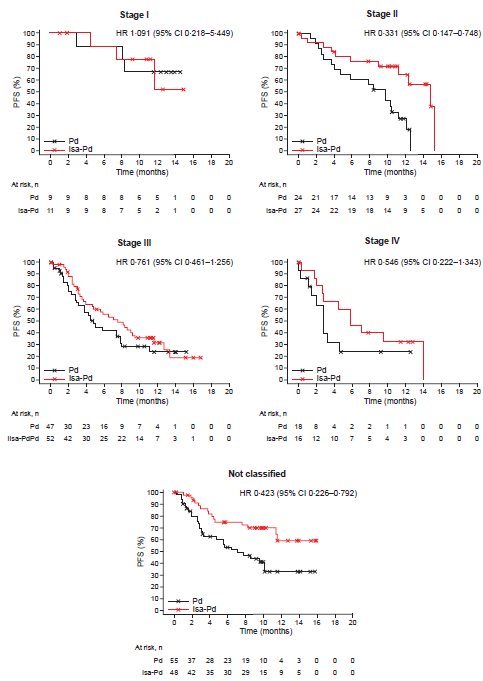


# Fig. S5 Overall survival (isatuximab–pomalidomide–dexamethasone vs. pomalidomide–dexamethasone) in ICARIA-MM, by R2-ISS stage. *CI* confidence interval, *HR* hazard ratio, *Isa-Pd* isatuximab–pomalidomide–dexamethasone, *OS* overall survival, *Pd* pomalidomide–dexamethasone, *R2-ISS* Second Revision of the International Staging System.


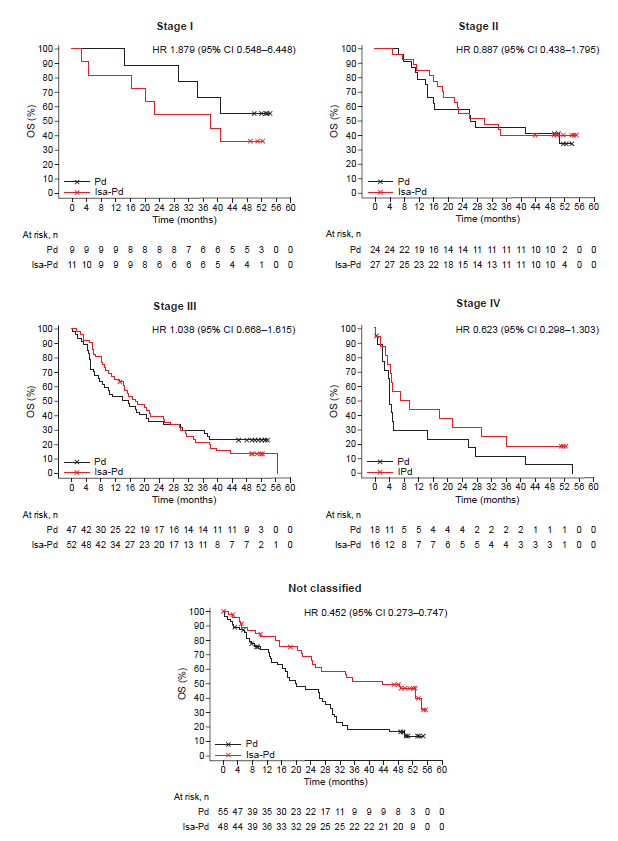


**Fig. S6 Progression-free survival (isatuximab–carfilzomib–dexamethasone *vs* carfilzomib–dexamethasone) in IKEMA, by R2-ISS stage, based on disease assessment by the independent response committee.** *CI* confidence interval, *HR* hazard ratio, *Isa-Kd* isatuximab–carfilzomib–dexamethasone, *Kd* carfilzomib–dexamethasone, *PFS* progression-free survival, *R2-ISS* Second Revision of the International Staging System.
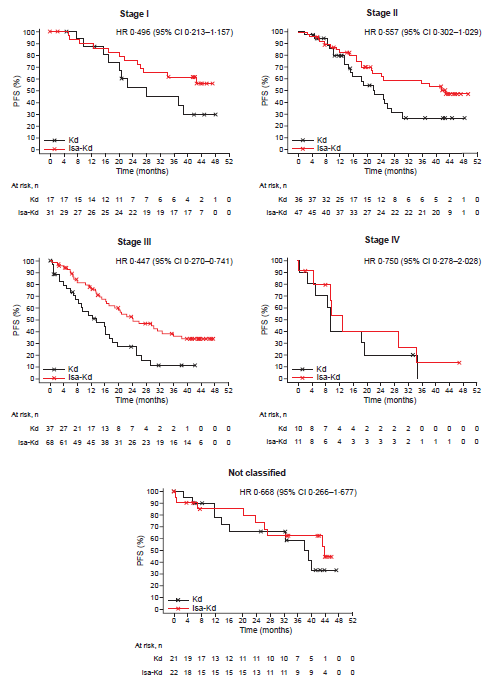


# Fig. S7 Overall survival (isatuximab–carfilzomib–dexamethasone *vs* carfilzomib–dexamethasone) in IKEMA, by R2-ISS stage. *CI* confidence interval, *HR* hazard ratio, *Isa-Kd* isatuximab–carfilzomib–dexamethasone, *OS* overall survival, *R2-ISS* Second Revision of the International Staging System.


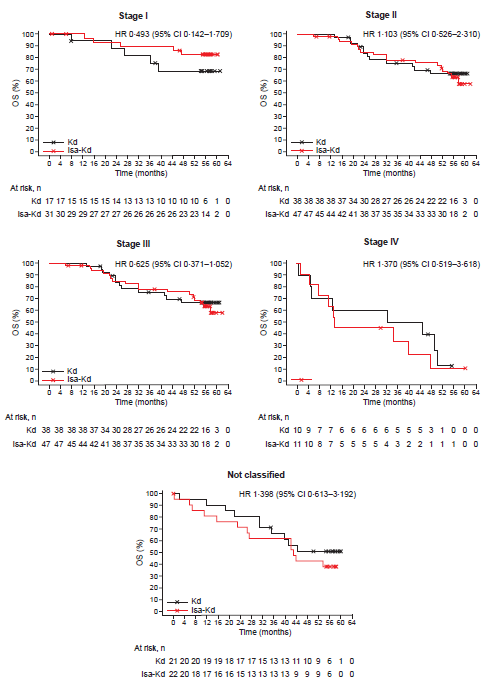


# Table S3. Progression-free survival by R2-ISS stage versus R2-ISS stage 1 (pooled early relapse populations from ICARIA-MM and IKEMA).

|  | **R2-ISS stage** | | | |
| --- | --- | --- | --- | --- |
|  | **Stage I**  **(N = 21)** | **Stage II (N = 51)** | **Stage III (N = 114)** | **Stage IV (N = 35)** |
| Number (%) of events | 6 (28.6) | 29 (56.9) | 77 (67.5) | 23 (65.7) |
| Number (%) of patients censored | 15 (71.4) | 22 (43.1) | 37 (32.5) | 12 (34.3) |
| Kaplan-Meier estimate in months |  |  |  |  |
| 25% quantile (95% CI) (months) | 26.3 (5.65–NC) | 7.6 (2.76–13.24) | 3.1 (2.43–4.67) | 2.8 (1.18–3.29) |
| Median survival (95% CI) (months) | NC (16.99–NC) | 16.9 (12.06–24.18) | 7.9 (5.75–11.53) | 4.6 (2.83–9.23) |
| 75% quantile (95% CI) (months) | NC (37.45–NC) | 24.8 (18.46–NC) | 24.1 (13.63–30.16) | 12.9 (4.99–34.53) |
| Stratified log-rank test *p*-value |  |  |  |  |
| vs. R2-ISS stage I |  | 0.0046 | <0.0001 | <0.0001 |
| Adjusted^a^ hazard ratio (95% CI) |  |  |  |  |
| vs. R2-ISS stage I |  | 3.41 (1.408–8.260) | 5.24 (2.261–12.125) | 7.33 (2.942–18.242) |

^a^Adjusted by treatment. CI confidence interval, NC not calculable, *R2-ISS* Second Revision of the International Staging System.

# Table S4. Progression-free survival (isatuximab-based triplet vs. doublet), by R2-ISS stage, among pooled early relapse populations from ICARIA-MM and IKEMA.

|  | **Triplet**  **(isatuximab–pomalidomide–dexamethasone,  isatuximab–carfilzomib–dexamethasone)** | | | **Doublet**  **(pomalidomide–dexamethasone,  carfilzomib–dexamethasone)** | | | **Hazard ratio (95% CI) triplet vs. doublet** |
| --- | --- | --- | --- | --- | --- | --- | --- |
|  | **N** | **n (%) of events** | **Median (months) (95% CI)** | **N** | **n (%) of events** | **Median (months) (95% CI)** |  |
| All patients^a^ | 154 | 84 (54.5) | 14.784 (9.232–24.181) | 140 | 85 (60.7) | 8.312 (5.585–12.057) | 0.624 (0.459–0.848) |
| R2-ISS stage |  |  |  |  |  |  |  |
| Stage I | 11 | 3 (27.3) | NC (7.425–NC) | 10 | 3 (30.0) | 37.454 (8.049–NC) | 0.991 (0.199–4.935) |
| Stage II | 27 | 15 (55.6) | 18.464 (14.784–24.739) | 24 | 14 (58.3) | 13.240 (8.411–24.805) | 0.774 (0.372–1.613) |
| Stage III | 63 | 41 (65.1) | 9.133 (5.815–13.634) | 51 | 36 (70.6) | 7.425 (3.811–11.138) | 0.657 (0.418–1.034) |
| Stage IV | 17 | 12 (70.6) | 5.782 (2.760–12.879) | 18 | 11 (61.1) | 3.285 (1.971–9.298) | 0.723 (0.316–1.656) |
| Not classified | 36 | 13 (36.1) | 43.072 (7.622–NC) | 37 | 21 (56.8) | 5.651 (2.891–10.086) | 0.366 (0.175–0.764) |

^a^Adjusted by R2-ISS stage. *CI* confidence interval, *NC* not calculable, *R2-ISS* Second Revision of the International Staging System.

# Fig. S8 Progression-free survival (isatuximab-based triplet vs. doublet), by R2-ISS stage, among pooled early relapse populations from ICARIA-MM and IKEMA.


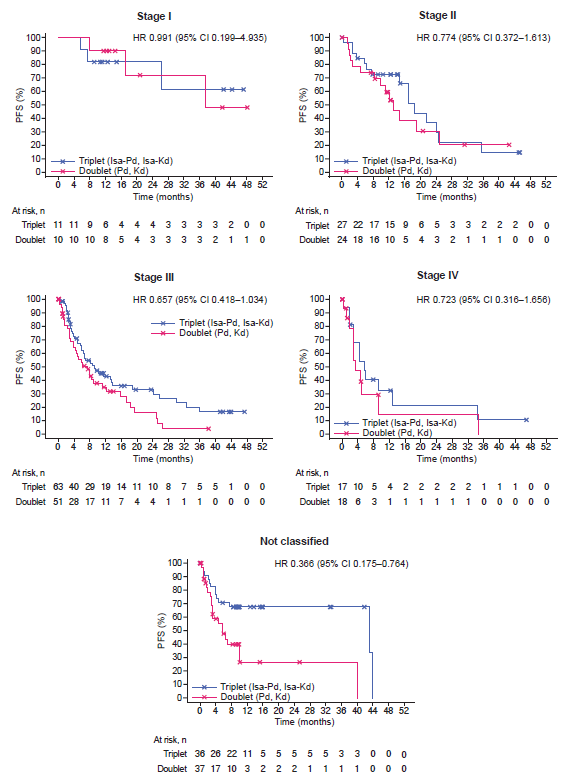


# Table S5. Overall survival (isatuximab-based triplet vs. doublet), by R2-ISS stage, among pooled early relapse populations from ICARIA-MM and IKEMA.

|  | **Triplet**  **(isatuximab–pomalidomide–dexamethasone,  isatuximab–carfilzomib–dexamethasone)** | | | **Doublet**  **(pomalidomide–dexamethasone,  carfilzomib–dexamethasone)** | | | **Hazard ratio (95% CI) triplet vs. doublet** |
| --- | --- | --- | --- | --- | --- | --- | --- |
|  | **N** | **n (%) of events** | **Median (months) (95% CI)** | **N** | **n (%) of events** | **Median (months) (95% CI)** |  |
| All patients^a^ | 154 | 107 (69.5) | 31.113 (23.031–37.651) | 140 | 103 (73.6) | 22.702 (14.982–29.733) | 0.755 (0.590–1.018) |
| R2-ISS stage |  |  |  |  |  |  |  |
| Stage I | 11 | 3 (27.3) | NC (20.271–NC) | 10 | 4 (40.0) | NC (14.390–NC) | 0.706 (0.158–3.160) |
| Stage II | 27 | 17 (63.0) | 42.382 (21.782–57.992) | 24 | 11 (45.8) | NC (19.187–NC) | 1.431 (0.669–3.062) |
| Stage III | 63 | 47 (74.6) | 25.232 (14.456–32.394) | 51 | 43 (84.3) | 17.117 (8.674–29.733) | 0.754 (0.498–1.142) |
| Stage IV | 17 | 15 (88.2) | 9.593 (3.515–34.398) | 18 | 17 (94.4) | 5.027 (3.745–14.357) | 0.748 (0.371–1.506) |
| Not classified | 36 | 25 (69.4) | 42.251 (24.181–48.197) | 37 | 28 (75.7) | 17.544 (10.086–31.080) | 0.608 (0.352–1.048) |

^a^Adjusted by R2-ISS stage. *CI* confidence interval, *NC* not calculable, *R2-ISS* Second Revision of the International Staging System.

# Fig. S9 Overall survival (isatuximab-based triplet vs. doublet), by R2-ISS stage, among pooled early relapse populations from ICARIA-MM and IKEMA.

**
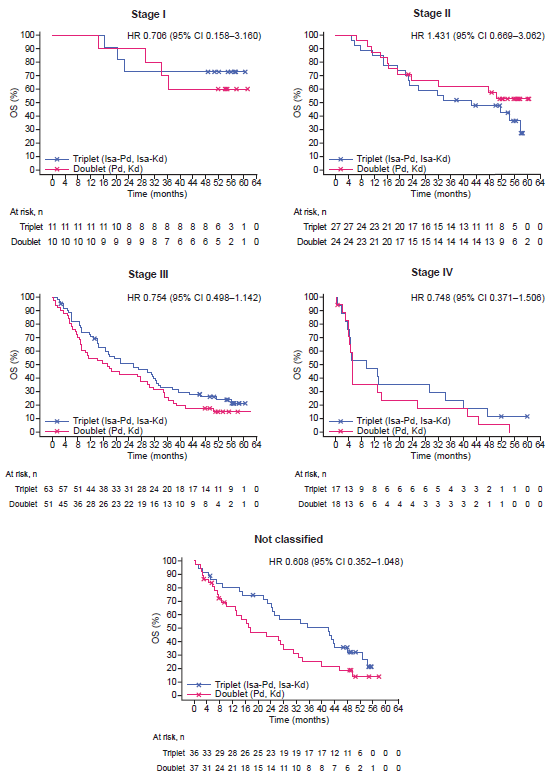
**

# Table S6. Progression-free survival by R2-ISS stage versus R2-ISS stage I (pooled intention-to-treat populations from ICARIA-MM and IKEMA) without allowance for missing data.

|  | **R2-ISS stage** | | | |
| --- | --- | --- | --- | --- |
|  | **Stage I**  **(n = 68)** | **Stage II (n = 136)** | **Stage III (n = 191)** | **Stage IV (n = 51)** |
| Number (%) of events | 28 (41.2) | 71 (52.2) | 119 (62.3) | 35 (68.6) |
| Number (%) of patients censored | 40 (58.8) | 65 (47.8) | 72 (37.7) | 16 (31.4) |
| Kaplan-Meier estimate in months |  |  |  |  |
| 25% quantile (95% CI) (months) | 17.0 (8.57–22.44) | 10.3 (7.85–12.25) | 4.7 (3.09–6.01) | 2.8 (1.45–4.57) |
| Median survival (95% CI) (months) | 38.8 (22.44–NC) | 21.2 (15.21–25.99) | 12.2 (8.94–16.16) | 8.7 (4.44–9.72) |
| 75% quantile (95% CI) (months) | NC (NC–NC) | NC (38.11–NC) | 29.2 (20.80–NC) | 18.5 (9.49–34.53) |
| Stratified log-rank test *p*-value |  |  |  |  |
| vs. R2-ISS stage I |  | 0.037 | <0.0001 | <0.0001 |
| Adjusted^a^ hazard ratio (95% CI) |  |  |  |  |
| vs. R2-ISS stage I |  | 1.53 (0.983–2.368) | 2.65 (1.747–4.027) | 3.39 (2.040–5.623) |

^a^Adjusted by treatment. *CI* confidence interval, *NC* not calculable, *R2-ISS* Second Revision of the International Staging System.

# Table S7. Overall survival by R2-ISS stage versus R2-ISS stage I (pooled intention-to-treat populations from ICARIA-MM and IKEMA) without allowance for missing data.

|  | **R2-ISS stage** | | | |
| --- | --- | --- | --- | --- |
|  | **Stage I**  **(n = 68)** | **Stage II (n = 136)** | **Stage III (n = 191)** | **Stage IV (n = 51)** |
| Number (%) of events | 20 (29.4) | 53 (39.0) | 126 (66.0) | 40 (78.4) |
| Number (%) of patients censored | 48 (70.6) | 83 (61.0) | 65 (34.0) | 8 (21.6) |
| Kaplan-Meier estimate in months |  |  |  |  |
| 25% quantile (95% CI) (months) | 38.8 (22.67–NC) | 23.7 (18.92–29.93) | 10.7 (8.31–14.23) | 4.1 (2.00–5.03) |
| Median survival (95% CI) (months) | NC (47.18–NC) | NC (50.27–NC) | 27.5 (21.55–32.69) | 12.9 (4.99–27.53) |
| 75% quantile (95% CI) (months) | NC (NC–NC) | NC (NC–NC) | 56.1 (NC–NC) | 39.9 (25.63–NC) |
| Stratified log-rank test *p*-value |  |  |  |  |
| vs. R2-ISS stage I |  | 0.2835 | <0.0001 | <0.0001 |
| Adjusted^a^ hazard ratio (95% CI) |  |  |  |  |
| vs. R2-ISS stage I |  | 1.31 (0.781–2.189) | 2.80 (1.741–4.496) | 4.03 (2.337–6.938) |

^a^Adjusted by treatment. *CI* confidence interval, *NC* not calculable, *R2-ISS* Second Revision of the International Staging System.

# REFERENCES

1. Richardson PG, Attal M, Campana F, Le-Guennec S, Hui AM, Risse ML, et al. Isatuximab plus pomalidomide/dexamethasone versus pomalidomide/dexamethasone in relapsed/refractory multiple myeloma: ICARIA Phase III study design. Future Oncol. 2018;14:1035-47.

2. Moreau P, Dimopoulos MA, Yong K, Mikhael J, Risse ML, Asset G, et al. Isatuximab plus carfilzomib/dexamethasone versus carfilzomib/dexamethasone in patients with relapsed/refractory multiple myeloma: IKEMA Phase III study design. Future Oncol. 2020;16:4347-58.

3. Richardson PG, Perrot A, San-Miguel J, Beksac M, Spicka I, Leleu X, et al. Isatuximab plus pomalidomide and low-dose dexamethasone versus pomalidomide and low-dose dexamethasone in patients with relapsed and refractory multiple myeloma (ICARIA-MM): follow-up analysis of a randomised, phase 3 study. Lancet Oncol. 2022;23:416-27.

4. Martin T, Dimopoulos MA, Mikhael J, Yong K, Capra M, Facon T, et al. Isatuximab, carfilzomib, and dexamethasone in patients with relapsed multiple myeloma: updated results from IKEMA, a randomized Phase 3 study. Blood Cancer J. 2023;13:72.
